# Radiomics on slice-reduced versus full-chest computed tomography for diagnosis and staging of interstitial lung disease in systemic sclerosis: A comparative analysis

**DOI:** 10.1016/j.ejro.2024.100596

**Published:** 2024-08-30

**Authors:** Anja A. Joye, Marta Bogowicz, Janine Gote-Schniering, Thomas Frauenfelder, Matthias Guckenberger, Britta Maurer, Stephanie Tanadini-Lang, Hubert S. Gabryś

**Affiliations:** a University Hospital of Zurich, Department of Radiation Oncology, Rämistrasse 100, Zürich 8091, Switzerland; b Center of Experimental Rheumatology, Department of Rheumatology, University Hospital Zurich, University of Zurich, Zurich, Switzerland; c University Hospital of Zurich, University Zurich, Institute for Diagnostic and Interventional Radiology, Switzerland

**Keywords:** Systemic sclerosis, Interstitial lung disease, Slice-reduced computed tomography, Radiomics, Machine learning, Medical image analysis

## Abstract

**Purpose:**

The purpose of this study was to evaluate the efficacy of radiomics derived from slice-reduced CT (srCT) scans versus full-chest CT (fcCT) for diagnosing and staging of interstitial lung disease (ILD) in systemic sclerosis (SSc), considering the potential to reduce radiation exposure.

**Material and methods:**

The fcCT corresponded to a standard high-resolution full-chest CT whereas the srCT consisted of nine axial slices. 1451 radiomic features in two dimensions from srCT and 1375 features in three dimensions from fcCT scans were extracted from 166 SSc patients. The study included first- and second-order features from original and wavelet-transformed images. We assessed the predictive performance of quantitative CT (qCT)-based logistic regression (LR) models relying on preselected features and machine learning workflows involving LR and extra-trees classifiers with data-driven feature selection. The area under the receiver operating characteristic curve (AUC) was used to estimate model performance.

**Results:**

The best models for diagnosis and staging ILD achieved AUC=0.85±0.08 and AUC=0.82±0.08 with srCT, and AUC=0.83±0.06 and AUC=0.76±0.08 with fcCT, respectively. srCT-based models showed slightly superior performance over fcCT-based models, particularly in 2D-radiomic analyses when interpolation resolution closely matched the original in-plane resolution. For diagnosis, the LR outperformed qCT-models, whereas for staging, the best results were obtained with a qCT-based model.

**Conclusions:**

Radiomics from srCT is an effective and preferable alternative to fcCT for diagnosing and staging SSc-ILD. This approach not only enhances predictive accuracy but also minimizes radiation exposure risks, offering a promising avenue for improved treatment decision support in SSc-ILD management.

## Highlights

• CT-based radiomics is suitable for diagnosis and staging interstitial lung disease in systemic sclerosis.
• Radiomics on slice-reduced CT provides superior results to radiomics on full-chest CT.
• Performance of models based on slice-reduced CT is sensitive to image resampling.
• Radiomics on slice-reduced CT offers an automated low-radiation diagnostic alternative to expert-graded full-chest CT scans.

## 1 Introduction

Interstitial lung disease (ILD) is a common complication of systemic sclerosis (SSc), a systemic multi-organ disorder [2]. As a leading cause of mortality among SSc patients, the early detection and effective management of ILD are critically important for improving patient outcomes and survival rates associated with this disease [3].

Since the highest risk of ILD occurs in the initial years post-SSc diagnosis, vigilant lung monitoring is essential. High-resolution computed tomography (HRCT) is currently the state-of-the-art technique for guiding prognostication and treatment decisions in SSc-ILD [3]. However, imaging with HRCT involves radiation exposure, which is a concern that limits the routine repetition of chest HRCT [4]. For this reason, its use as a screening tool in asymptomatic patients is not universally accepted [5]. One method to reduce the radiation dose while maintaining diagnostic accuracy is to perform limited, interspaced data acquisition [6]. This involves a dedicated, reduced HRCT protocol consisting of only a few slices allocated according to a basal-apical gradient.

As ILD is a disease with a highly variable course, treatment decisions need to be patient-specific [1]. This need for highly individualized treatment can be met by precision medicine approaches using quantitative analysis of medical images [7]. Radiomics, with its high-throughput extraction of imaging features, can quantify tissue properties beyond visual interpretation or simple quantitative CT analysis. Radiomic features, together with data mining and machine-learning tools, facilitate development of predictive models for various clinical outcomes [8]. Initially, radiomics was applied to describe tumor phenotypes in order to predict diagnosis, treatment response or prognosis [9]. However, radiomics has also been successfully applied in non-tumorigenic research [10], [11] and lung disease imaging [12].

Radiomics has shown potential in ILD to support clinical decision making. It has been effective in classifying the GAP stage (gender, age, and pulmonary function) in SSc-ILD [13] and predicting ILD occurrence [14]. It has also been applied to microCT scans of ILD in mice, with findings translating to HRCT of SSc-ILD in humans [14]. Further, quantitative CT analysis and radiomics have been used to assess ILD severity and pulmonary function tests [15]. Studies have shown the radiomics’ potential for risk stratification and outcome prediction for progression-free survival in SSc-ILD [16]. Additionally, radiomics may predict mortality in patients with rheumatoid arthritis and ILD [17]. Lung graph-based radiomic models achieved high accuracy in identifiying fibrotic ILD [18]. However, to our knowledge, no study has investigated the predictive power of radiomics on disease detection and extent according to the Goh score, comparing srCT with fcCT.

In this study, we compared radiomic features extracted from full-chest CT (fcCT) and slice-reduced CT (srCT) scans in terms of interchangeability and discriminative power. We aimed to evaluate whether srCT-based radiomic models can serve as an effective substitute for traditional fcCT, potentially offering a safer yet equally informative, diagnostic tool in the management of SSc-ILD.

## 2 Material and methods

### 2.1 Patient Cohort

The underlying retrospective patient cohort for this study consisted of 170 patients diagnosed with SSc (Table 1) and was adopted from a previous prospective study [6]. Four patients were excluded due to retracted general consent. For the remaining 166 patients, a standardized full-chest HRCT scan and a slice-reduced HRCT scan taken on the same date, as well as clinical data (age, sex, disease status (GOH)), were available. The diagnostic criteria and severity of lung disease were based on the Goh score [19]. According to the Goh scoring system, the extent of ILD is divided into three categories: no lung involvement, limited (disease extent on HRCT <20 %) and extensive (>20 %) involvement. The scoring was performed by an experienced radiologist (TF). The local ethics committees approved the study (approval numbers: pre-BASEC-EK-839 (KEK-no.–2016–01515), KEK-ZH-no. 2010–158/5, BASEC-no. 2018–02165, BASEC-no. 2018–01873) and general consent was obtained from every patient.

**Table 1:** Patient characteristics. Patient position: feet-first-prone (FFP), head-first-prone (HFP) or feet-first-supine (FFS).

		**All**	**Fibrosis stage**		
			none	limited	extensive
Total patients		166	99	44	23
Age (years)	median	57	55	58	63
	Q1-Q2	48–66	47–64	49–66	55–67
	range	16–82	16–82	22–76	40–82
Sex	female	136	86	33	17
	male	30	13	11	6
**Full-chest CT scans:**					
Patient position	FFP	146	92	37	17
	HFP	14	7	5	2
	FFS	6	-	2	4
CT reconstruction kernel	B60f	18	9	4	5
	B70f	145	90	40	15
	BL64d	3	-	-	3
Tube voltage	120 kVP	161	99	77	18
	100 kVP	5	-	-	5
Tube current (mAs)	range	78–684	85–684	78–553	94–647
Slice thickness	1 mm 2 mm 0.6 mm	163 2 1	99 - -	44 - -	20 2 1
Pixel spacing (mm)	range	0.48–0.86	0.51–0.77	0.48–0.86	0.55–0.84
**Slice-reduced CT scans:**					
Patient position	FFP	149	92	37	20
	HFP	14	7	5	2
	FFS	3	-	2	1
CT reconstruction kernel	B70s	92	59	26	7
	B80s	71	40	18	13
	BL57s	3	-	-	3
Tube voltage	120 kVP	163	99	44	20
	100 kVP	3	-	-	3
Tube current (mAs)	range	18–272	18–248	18–272	23–190
Slice thickness (mm)	1 mm	166	99	44	23
Pixel spacing (mm)	range	0.53–0.82	0.53–0.82	0.55–0.82	0.59–0.72

### 2.2 Imaging Data

CT images were acquired in full inspiration with three types of scanners: 128-slice multidetector CT scanner (Siemens Somatom Definition Flash), 64-slice multidetector CT scanner (Siemens Somatom Definition AS) and 192-slice multidetector CT scanner (Siemens Somatom Force). Raw imaging data was reconstructed using filtered back projection.

**Fig. 1:**
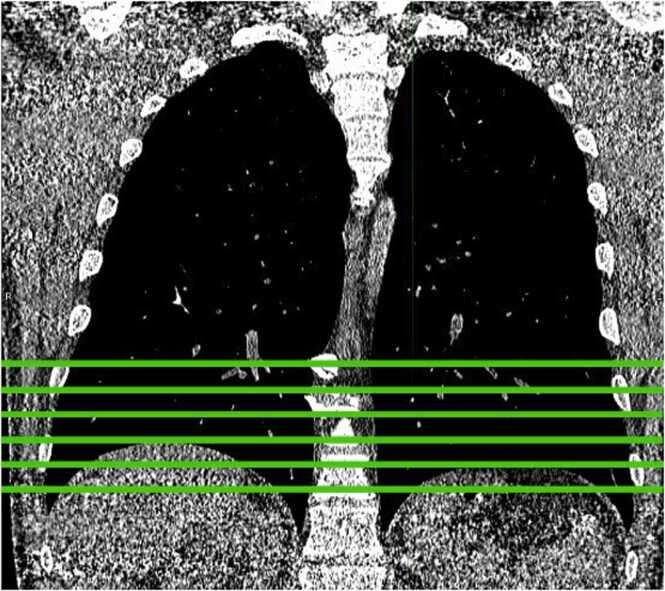
Position of six bottom slices acquired with an increment of 15 mm in srCTs.

For the srCT protocol, nine sequential slices were acquired with a basal-apical gradient, consistent with the mainly basally located parenchymal changes in SSc-ILD. In fact, the nine-slice CT scan comprised two sub-scans; one consisting of three upper slice positions and one of six bottom slice positions. For some patients, the lowest slice from the upper scan had a lower slice position than the highest slice from the lower scan or the highest slice from the upper scan was not situated in the lung. For further analysis, only the six bottom slice positions were considered. This ensured a basal-apical gradient of all slices with an identical spacing of 15 mm for all patients. Furthermore, it was assumed that the three slices from the upper scan do not provide much additional information for SSc-ILD prediction since most SSc patients develop ILD starting at the bottom of the lung. Therefore, it was expected that discarding the upper scan would lead to better predictions of SSc-ILD. Therefore, for further analysis, only the six bottom slice positions were considered (Fig. 1). Furthermore, all positions of the reduced CT scan consisted of two slices. This was due to technical limitations of CT scanners. Some of these slices showed different levels of image noise. Therefore, for each slice position, the slice with less noise was taken for further analysis. As measure for the noise level, the signal-to-noise ratio was chosen.

### 2.3 Extraction of Radiomic Features

**Fig. 2:**
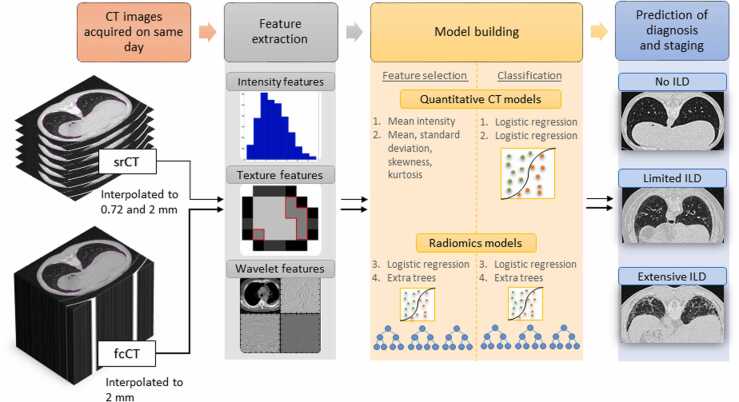
A flowchart visualizing the analysis process. Boths lungs as regions of interest (ROI) were segmented on srCT (slice-reduced CT) and fcCT (full-chest CT). Radiomic features were extracted from the ROIs on interpolated images, including intensity, texture and wavelet features. Four different predictive models have been built with different feature selection and classification methods to predict ILD diagnosis and stage.

Both lungs, as the region of interest for the following radiomics analysis, were semi-automatically segmented on the fcCTs and srCTs. An in-house developed radiomics software, Z-Rad, was used for preprocessing and calculation of radiomic features from the contoured CT images in agreement with the Image Biomarker Standardization Initiative [20]. Radiomic features from srCTs and fcCTs were extracted in two-dimensions (2D) and three-dimensions (3D), respectively (see Fig. 2).

Preprocessing steps applied prior to the radiomic feature extraction were voxel/pixel resampling and grey-level discretization. fcCTs were interpolated to an isotropic voxel size of 2 mm. For srCTs, images were resampled in-plane to a pixel size of 0.72 and 2 mm. A resolution of 0.72 mm was chosen for the interpolation to minimize effects of up-sampling and to represent original images best without introducing artificial in-plane information. The additional resolution of 2 mm was chosen to facilitate comparison between 2D- and 3D-radiomic features by minimizing the influence of different interpolation resolutions. For grey-level discretization, a fixed bin width of 50 Hounsfield units (HU) was chosen, ranging from −1000 HU to 200 HU.

Radiomics calculation delivered a total of 1451 2D-radiomic features for srCTs with 2 & 0.72 mm interpolation resolution, respectively, and 1375 3D-radiomic features for fcCTs. The extracted features quantified lung characteristics and can be divided into three types, such as intensity histogram, texture, and wavelet features. Features with missing values or counting less than five unique values were removed from further analysis. A list of extracted features is provided in the Supplement A1.

### 2.4 Statistical Methods

A univariate analysis was performed to investigate associations of single features with the patient’s disease stage. The area under the receiver operating characteristic curve (AUC) of individual features was calculated using the *U* statistic [21], [22].

Two types of multivariate models were evaluated in this study: models with preselected features and machine learning models with a data-driven feature selection procedure. The models with preselected features allowed us to establish a performance baseline and compare the results to other qCT studies. The machine learning models were used to explore a broad domain of radiomic features and more complex classification patterns.

Two qCT logistic regression models with preselected features were considered. The 'M' model was based on the mean of the intensity distribution (M), which captures the average value of the image intensities. The 'MSDSK' model was based on the mean, standard deviation, skewness, and kurtosis of the intensity distribution (MSDSK), which provides a more comprehensive information by including the average value, variability, asymmetry, and tailedness of the image intensities.

We considered two machine learning workflows to build data-driven models in this study: 1) logistic regression with embedded logistic regression-based feature selection (LR) and 2) Extra-Trees with embedded Extra-Trees-based feature selection (ET). Before feature selection with these embedded methods, features were preprocessed. Features were selected according to the Pearson’s *r* with a tunable cut-off value. Correlated features with a coefficient above the cutoff value were removed according to their rank. The ranking method was subject to hyperparameter tuning and was either the order in which the features were extracted (intensity, texture and then wavelets), according to the *F*-score or according to the Kruskal-Wallis *H* test statistic value. Then, features were scaled and transformed using the *Z*-score feature normalization followed by the Yeo-Johnson power transform [23].

A nested cross-validation (nCV) was used to train, optimize, and test the models. The inner loop, used for model tuning, was a 3x-repeated 10-fold cross-validation with 300 randomly generated hyperparameter samples that were used for random search optimization [24]. The outer loop, used for model testing, was a 2x-repeated 5-fold cross-validation. The performance metric used for the model optimization was the AUC. The optimal cutoff, corresponding to the maximal value of Youden's index [25], was calculated for every model from the averaged receiver operating characteristics (ROC) curves of the nCV outer loop. Based on the optimal cutoff, accuracy, sensitivity, specificity, predictive values, and likelihood ratios with corresponding confidence intervals (CI) were estimated. For additional inspection of model performance, the precision-recall (PR) curves were used, as the classes were imbalanced. Feature selection methods as well as classification methods and their parameter configurations for hyperparameter optimization are listed in the Supplement A2.

The model building, visualization, and statistical analysis was conducted in Python using following open-source packages: Matplotlib [26], Seaborn [27], NumPy [28] & SciPy [29], Pandas [30], scikit-learn [31].

## 3 Results

### 3.1 Univariate association of features with the disease state

**Fig. 3:**
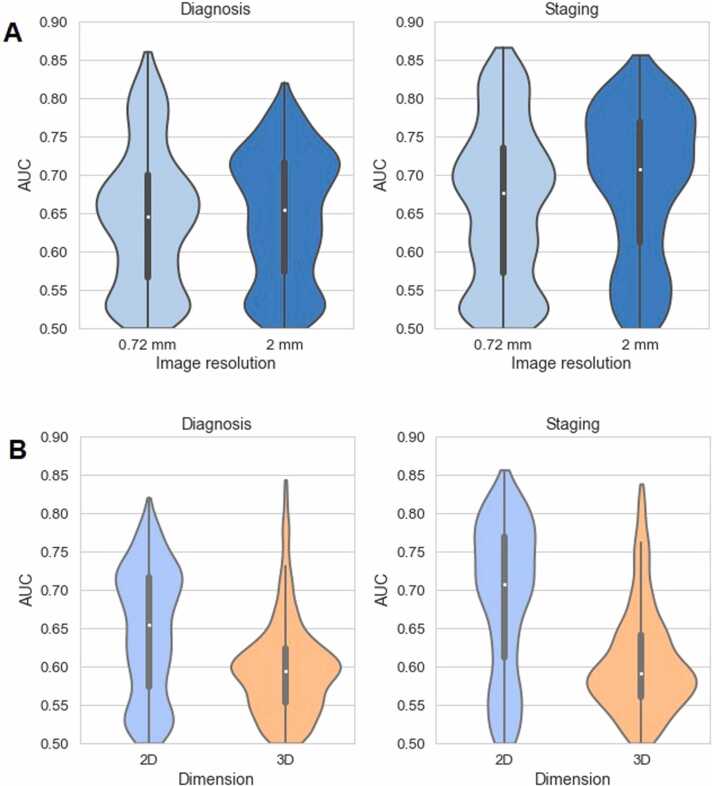
Distribution of predictive power (AUC) of features. A): AUC distribution of features extracted in 2D (0.72 mm and 2 mm resolution). B): AUC distribution of features extracted in 2D and 3D (both 2 mm interpolation resolution): The AUC scores less than 0.5 corresponded to features in which high feature values were more likely to occur in the negative class. Such AUCs were subtracted from 1 to facilitate comparison of all features. This allowed analyzing the strength of general predictive power, independent of the direction of the effect.

The distribution of AUC scores, the probability, that the model will rank a randomly chosen positive instance higher than a randomly chosen negative one, is presented in Fig. 3. In 2D-radiomics, the median values were slightly higher for 2 mm resolution for both diagnosis and staging. This indicates that more features with a highly predictive performance were extracted from images interpolated to 2 mm resolution. However, features from 0.72 mm resolution showed the highest observed AUC scores.

To analyze predictive power of features in 2D versus 3D, corresponding distributions of AUC scores were compared against each other for 2 mm interpolation resolution. Univariate predictive power of radiomic features extracted from fcCT was on average lower compared to srCT. However, the distributions of 2D-radiomics AUC scores were considerably more spread. For diagnosis, 3D-radiomics delivered the highest predictive features compared to 2D-radiomics. For staging, the maximal AUC scores were slightly higher for features extracted in 2D.

### 3.2 Multivariate models predicting diagnosis and stage of SSc-ILD

The best performing multivariate models were both obtained from srCT in 2D. The best diagnostic model used features extracted from images with a 0.72 mm resolution, utilizing logistic regression for both feature selection and classification. This model achieved an AUC of 0.85±0.08. For 84 % of patients (139 out of 166), the predicted diagnosis was correct. The best staging model was an MSDSK model based on 0.72 mm resolution images, with an AUC of 0.82±0.08. For 79 % of patients (53 out of 67), the predicted disease stage was correct. Predictive performance of the models is shown in Table 2.

**Table 2:** Model performance in terms of AUC scores estimated with nested CV. Higher AUC values indicate better model performance in distinguishing between the positive (ILD / extensive disease) and negative (no ILD / limited disease). Feature selection was either predefined (mean intensity (M) and mean, standard deviation, skewness, and kurtosis (MSDSK) of the intensity distribution) or based on models (select from model (SFM): logistic regression (LR) and extra trees (ET)). Classifier: logistic regression (LR) or extra trees (ET). The best models in 2D and 3D for diagnosis and staging are in bold print.

**Model**			**Diagnosis**		**Staging**	
Feature calculation	Feature Selection	Classifier	AUC Tuning	AUC Testing	AUC Tuning	AUC Testing
2D (0.72 mm)	M	LR	0.773 ± 0.108	0.774 ± 0.059	0.796 ± 0.192	0.786 ± 0.097
	MSDSK	LR	0.808 ± 0.131	0.800 ± 0.084	0.814 ± 0.185	**0.819 ± 0.083**
	LR	LR	0.864 ± 0.115	**0.851 ± 0.078**	0.832 ± 0.231	0.784 ± 0.090
	ET	ET	**0.866 ± 0.117**	0.828 ± 0.063	**0.833 ± 0.202**	0.712 ± 0.149
2D (2 mm)	M	LR	0.768 ± 0.115	0.770 ± 0.069	0.790 ± 0.202	0.782 ± 0.098
	MSDSK	LR	0.784 ± 0.128	0.778 ± 0.089	**0.819 ± 0.197**	**0.811 ± 0.090**
	LR	LR	0.834 ± 0.113	**0.804 ± 0.083**	0.802 ± 0.224	0.755 ± 0.100
	ET	ET	**0.840 ± 0.106**	0.792 ± 0.098	0.819 ± 0.218	0.760 ± 0.055
3D (2 mm)	M	LR	0.682 ± 0.117	0.683 ± 0.048	0.736 ± 0.215	0.720 ± 0.121
	MSDSK	LR	0.762 ± 0.119	0.763 ± 0.043	0.757 ± 0.239	0.706 ± 0.142
	LR	LR	0.866 ± 0.079	**0.825 ± 0.057**	0.820 ± 0.177	0.724 ± 0.107
	ET	ET	**0.871 ± 0.088**	0.822 ± 0.064	**0.835 ± 0.193**	**0.761 ± 0.081**

The predefined qCT models (M and MSDSK) performed better on srCT rather than on fcCT for both diagnosis and staging. The best performing predefined model was obtained with a MSDSK-model in 2D based on images with 0.72 mm resolution. For diagnosis, using radiomic models led to higher predictive performances than using handcrafted models. Corresponding generalization AUC scores were slightly higher for 2D- than 3D-radiomics. Models built on features extracted from images interpolated to a resolution of 0.72 mm in 2D showed the highest AUC scores. For staging, using a more complex model did not always result in higher predictive performance. Even though the AUC scores typically increased in tuning with applying more complex models, generalization AUC scores from nCV did not show the same pattern.

**Fig. 4:**
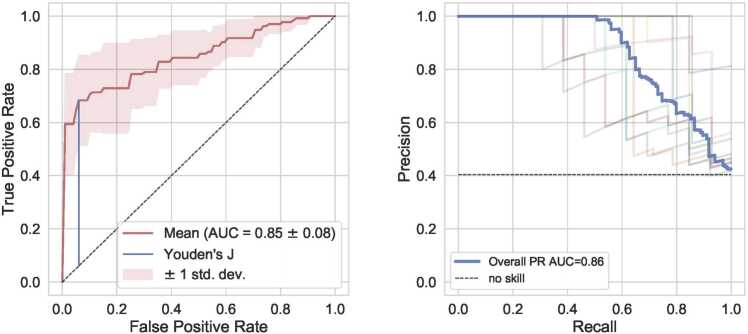
ROC and PR curves for the performing model: predicting diagnosis with AUC = 0.85 ± 0.08.

**Fig. 5:**
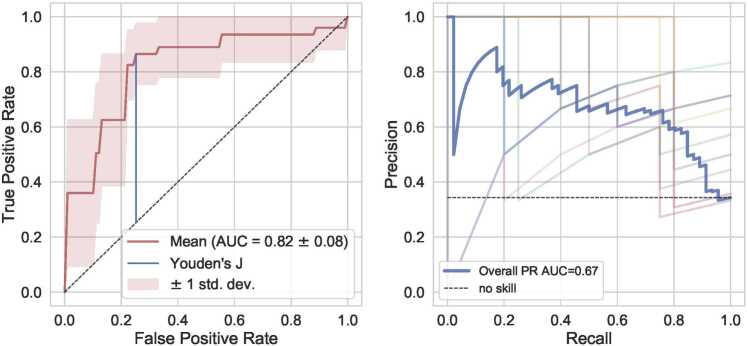
ROC and PR curves for the performing model: predicting disease stage with AUC = 0.82 ± 0.08.

The ROC and PR curves of the best performing model for diagnosis and staging are shown in Fig. 4 and Fig. 5, respectively (more in Supplement A3). The PR curves show the trade-off between precision (ratio of true positives to all positive predictions) and recall (the ratio of true positives to all actual positives). The area under the PR curve (PR AUC) indicates overall model performance, with higher values signifying better performance. In the ROC curve, the maximum Youden’s index is visualized. Further evaluation metrics (Table 3) were determined based on the location of maximum Youden’s index. The best diagnostic model (2D (0.72 mm) – LR – LR) showed the highest performance over all built models for diagnosis as well as staging. Higher values in *GLCM-sumVariance* and *GLSZM-zsEntropy* features and lower values in the *LL-GLCM-clusterShade* feature indicated higher probability for classification as ILD. For the best staging model (2D (0.72 mm) – MSDSK - LR), the *mean*, *skewness* and *kurtosis* features showed a negative correlation whereas the *standard deviation* feature (*hist_sd*) covered most range and correlated with extensive disease.

**Table 3:** Evaluation metrics of predictive performances at a decision threshold at maximum Youden’s J for diagnosis and staging (with 95 % confidence intervals). Predictive values: positive predictive values (PPV) and negative predictive values (NPV).

	**Model**		**Accuracy**	**Classification probabilities**		**Predictive values**		**Diagnostic likelihood ratios (DLR)**	
	**Type**	**Name**		**Sensitivity**	**Specificity**	**PPV**	**NPV**	**DLR+**	**DLR-**
Diagnosis	2D (0.72 mm)	LR-LR	0.84 (0.77–0.90)	0.68 (0.57–0.79)	0.94 (0.89–0.99)	0.88 (0.80–0.97)	0.81 (0.74–0.89)	11.28 (5.11–24.91)	0.34 (0.24–0.48)
	2D (2 mm)	LR-LR	0.75 (0.67–0.83)	0.69 (0.58–0.80)	0.79 (0.71–0.87)	0.69 (0.58–0.80)	0.79 (0.71–0.87)	3.26 (2.16–4.93)	0.39 (0.27–0.57)
	3D (2 mm)	LR-LR	0.82 (0.76–0.89)	0.64 (0.52–0.75)	0.95 (0.91–0.99)	0.90 (0.81–0.98)	0.79 (0.72–0.87)	12.60 (5.26–30.16)	0.38 (0.28–0.53)
Staging	2D (0.72 mm)	MSDSK-LR	0.79 (0.68–0.90)	0.87 (0.73–1.00)	0.75 (0.62–0.88)	0.64 (0.47–0.81)	0.91 (0.82–1.01)	3.43 (2.01–5.84)	0.18 (0.06–0.52)
	2D (2 mm)	MSDSK-LR	0.77 (0.65–0.88)	0.80 (0.64–0.96)	0.75 (0.62–0.88)	0.62 (0.45–0.80)	0.88 (0.77–0.98)	3.17 (1.83–5.48)	0.27 (0.12–0.62)
	3D (2 mm)	ET-ET	0.75 (0.63–0.87)	0.76 (0.59–0.93)	0.75 (0.62–0.88)	0.61 (0.43–0.79)	0.86 (0.75–0.97)	3.01 (1.72–5.26)	0.32 (0.15–0.68)

## 4 Discussion

Visual fcCT analysis is a standard method to evaluate the severity and evolution of ILD. Nevertheless, visual assessment is relatively insensitive to slight changes or early disease and is subject to intra- and interobserver variability even among experts [12]. Radiomics, a quantitative imaging tool, enhances visual assessments by providing imaging biomarkers [32]. This study compared radiomics on srCT versus fcCT for diagnosing and staging SSc-ILD, highlighting the potential of srCT to reduce radiation exposure without sacrificing diagnostic integrity.

As first step in this study, we considered two qCT models with preselected features to establish baseline performance and compare them to other qCT studies: 1) a logistic regression model based on the mean of the intensity distribution (M) and 2) a logistic regression model based on the mean, standard deviation, skewness, and kurtosis of the intensity distribution (MSDSK). The identified association of intensity-based features with ILD state was in line with results reported by other studies. Several publications have demonstrated that qCT measures (basic density histogram-based features) are related to ILD [33], [34], [35], [36], [37]. Bocchino et al. proposed a qCT index for SSc-ILD diagnosis using low-dose volumetric HRCT reporting AUC (95 % CI) of 0.77 (0.67–0.88) [38]. Our M-model achieved similar scores (AUC=0.77±0.06), whereas our MSDSK-model performed even better (AUC=0.80±0.08). Remarkably, the qCT models based on srCT outperformed these based on fcCT, suggesting that focusing on the ILD-prone lower lung regions can capture essential diagnostic information.

Although the qCT features can be easily computed, they rely on lung density, which can be heavily influenced by lung volume [39]. In the next step of this study, we applied machine-learning workflows to explore a broad domain of radiomic features and more complex classification patterns. Previous studies highlighted the radiomics’ potential in SSc-ILD for risk stratification, outcome prediction [16] and for classification of the GAP stage (gender, age, and pulmonary function) [13]. However, to our best knowledge, no study investigated the predictive power of radiomics on disease detection and extent according to the Goh score, comparing srCT with fcCT.

For SSc-ILD diagnosis, radiomic models led to higher predictive performance than the qCT models. The best result was obtained with feature selection and classification based on LR in 2D with 0.72 mm resolution (AUC=0.85±0.08). For SSc-ILD staging, radiomic-based models increased predictive performance in 3D but not in 2D. The best performing model predicting the disease stage was the predefined MSDSK-model from srCT images with 0.72 mm resolution (AUC=0.82±0.08). The slightly worse performance of the radiomic models in 2D may indicate the need for more data for modeling of the disease stage, as more complex and flexible models usually require more data to recognize the patterns in data and to correctly learn the relationship between the inputs and the outcome [40].

For both diagnosis and staging, the models obtained from srCT scans performed similar or slightly better than models obtained from fcCT scans. This finding seems to be counter-intuitive, as by acquiring srCT scans a lot of information about the lung is discarded. However, the srCT focuses on the lower part of the lung where the presence of SSc-ILD is usually most pronounced [41]. That way, a higher density of information (diseased tissue) is captured in srCTs. Thus, the characteristics of a lung affected with ILD are retained better in radiomic features extracted from the srCTs. fcCTs include the whole lung and therefore, have a larger proportion of healthy tissue than srCTs. In general, the srCT protocol allowed to increase signal-to-noise ratio in 2D features compared to 3D features which is reflected in higher univariate AUC scores and better performance of multivariate models. This aligns with other studies that have demonstrated how model calculations and radiomic features vary based on the spatial location of radiomics calculation, due to the spatial heterogeneities in ILD disease patterns [18], [42].

Comparing the dependence on the interpolation resolution in 2D, we found that a larger number of highly predictive features could be extracted with 2 mm resolution. However, features extracted from images with 0.72 mm resolution showed the highest AUC scores, suggesting that a higher resolution retains better critical tissue characteristics for detecting ILD. As a resolution of 0.72 mm was closer to the in-plane resolution of most CT scans in our cohort, effects from down-sampling could be kept to a minimum.

At present, studies on 2D- versus 3D-radiomics are limited. There are two studies investigating 2D- versus 3D-radiomics in ovarian cancer showing no significant difference between the predictive performance of 2D- versus 3D-radiomic models [43], [44]. Considering that the 2D model was cost-effective and time-efficient, the authors recommend using 2D features in future research [43].

In Frauenfelder et al. [6], two radiologists assessed ILD extent in SSc patients on srCT scans. Both radiologists recognized 68 of 77 diseased cases with an accuracy of 92 % and 95 % based on srCT. The accuracy of our best models was 84 % and 82 % for 2D- and 3D-radiomics, respectively. This indicates that radiomic models still need to be improved to reach human-level performance. Similarly to the radiologists in that study, our models might have missed the presence of ILD due to changes in the lung not captured on the reduced sampling protocol.

A significant advantage of this study is that radiomic analysis was based on fcCT and srCT scans acquired for each patient on the same day with the same CT scanner. This reduced variation in CT acquisition parameters and enabled an optimal radiomics comparison between the two CT protocols of a patient. Variations in image acquisition (e.g. dose, reconstruction algorithms) and processing parameters (e.g. different voxel sizes) can introduce noise and influence feature values, a common issue in radiomics [15].

There are a few limitations to this study. First, a single-center patient cohort might not be representative of the full variability of ILD-SSc, as disease differences due to patient demographics could be missed. Second, the fcCTs were resampled to a relatively large voxel size of 2 mm. The choice of this resolution was dictated by computational limitations stemming from large calculation volumes. Lastly, the models were validated only internally with nested cross-validation. Future external validation could strengthen the evidence supporting our models.

Our study showed that radiomics on srCT performs well, with no loss in predictive performance compared to radiomics on fcCT. Consequently, we suggest further investigation for its direct application in clinical practice. Frauenfelder et al. [6] provided evidence that the srCT protocol not only results in a much lower radiation burden but can also be implemented in daily clinical routine for early detection and screening of ILD. We recommend complementing the visual assessments of srCT with quantitative imaging biomarkers as an alternative objective method to support precision medicine approaches. Models could not only focus on diagnosis or staging of the disease but also on the prediction of disease progression. By doing so, a threshold for the initiation of treatment could be defined. Additionally, the calculation of radiomics in 2D versus 3D could be investigated on lung CT scans with a focus on other endpoints, such as ILD based on other diseases or pneumonia.

## 5 Conclusions

Applying radiomics in a srCT approach is a compelling alternative to fcCT. We showed that radiomic models based on srCT perform well for the diagnosis and staging of SSc-ILD. Our findings advocate for the broader adoption of medical image analysis using the srCT approach in clinical settings, emphasizing its dual advantages of significantly reducing radiation exposure, thereby enhancing patient safety, and promising higher predictive accuracy in radiomic analyses. Future applications could see such models integrated into clinical workflows, contributing significantly to the personalized management of SSc-ILD.

## Ethical

The local ethics committees approved the study (approval numbers: pre-BASEC-EK-839 (KEK-no.–2016–01515), KEK-ZH-no. 2010–158/5, BASEC-no. 2018–02165, BASEC-no. 2018–01873) and written informed consent was obtained from every patient.

## Funding

This work was supported by the Forschungskredit PostDoc from 10.13039/501100006447University of Zurich (FK-19–046 to Janine Gote-Schniering) and Swiss National Fund (SNF 310030_170159 to Hubert Gabrys).

## CRediT authorship contribution statement

**Anja A. Joye:** Writing – original draft, Visualization, Validation, Software, Methodology, Investigation, Formal analysis, Data curation. **Marta Bogowicz:** Writing – review & editing, Software. **Britta Maurer:** Writing – review & editing, Conceptualization. **Stephanie Tanadini-Lang:** Writing – review & editing, Validation, Supervision, Resources, Project administration, Conceptualization. **Hubert S. Gabrys:** Writing – original draft, Visualization, Validation, Supervision, Software, Methodology, Formal analysis, Conceptualization. **Janine Gote-Schniering:** Writing – review & editing. **Thomas Frauenfelder:** Writing – review & editing, Data curation. **Matthias Guckenberger:** Writing – review & editing, Validation, Supervision, Resources, Project administration.

## Declaration of Competing Interest

The authors declare the following financial interests/personal relationships which may be considered as potential competing interests:

We hereby state that the following authors have no conflicts of interest: Anja Alessandra Joye, Marta Bogowicz, Janine Gote-Schniering, Britta Maurer, Hubert Szymon Gabryś
The University Hospital Zurich has research and teaching agreements with Siemens Healthineers.
Thomas Frauenfelder: Speakers Bureau for Bracco and Bayer
Matthias Guckenberger: Siemens Healthineers, AstraZeneca
Stephanie Tanadini-Lang: Her husband is working at Siemens Healthineers
