# Supplementary material for: Radiomics on slice-reduced versus full-chest computed tomography for diagnosis and staging of interstitial lung disease in systemic sclerosis: A comparative analysis
Source: Eur J Radiol Open. 2024 Aug 30;13:100596. doi: 10.1016/j.ejro.2024.100596 (PMC11402420; doi:10.1016/j.ejro.2024.100596)
Supplement: Supplementary file 1 — Supplementary material. [file mmc1.docx]

# Supplement

### Extracted Features

| Feature type | Intensity-histogram | GLCM | NGTDM | GLRLM |
| --- | --- | --- | --- | --- |
| Feature name | minimum  maximum  mean  standard deviation  coefficient of variation  skewness  kurtosis  variance  median  percentile 10th  percentile 90th  interquartile range  range  mean absolut deviation  robust mean absolut deviation  energy  entropy  root mean square  uniformity | energy (angular second moment)  entropy  contrast  correlation  homogeneity  homogeneity normalized  inverse difference  inverse difference normalized  variance  sum of average  sum of entropy  sum of variance  difference entropy  difference variance  information measures of correlation 1  information measures of correlation 2  maximal correlation coefficient  joint maximum  joint average  difference average  dissimilarity  inverse variance  autocorrelation  cluster tendency  cluster shade  cluster prominence | coarseness  contrast  busyness  complexity  strength | gray-level non-uniformity  gray-level non-uniformity normalized  run length non-uniformity  run length non-uniformity normalized  short run emphasis  long runs emphasis  low gray-level run emphasis  high gray-level run emphasis  short run low gray-level emphasis  short run high gray-level emphasis  long run low gray-level emphasis  long run high gray-level emphasis  run percentage  gray level variance  run length variance  run entropy |

| Feature type | GLSZM | GLDZM | NGLDM |
| --- | --- | --- | --- |
| Feature name | gray-level non-uniformity  gray-level non-uniformity normalized  zone size non-uniformity  zone size non-uniformity normalized  small zone emphasis  large zone emphasis  low gray-level zone emphasis  high gray-level zone emphasis  small zone low gray-level emphasis  small zone high gray-level emphasis  large zone low gray-level emphasis  large zone high gray-level emphasis  zone percentage  gray level variance  zone size variance  zone size entropy | gray-level non-uniformity  gray-level non-uniformity normalized  zone distance non-uniformity  zone distance non-uniformity normalized  small distance emphasis  large distance emphasis  low gray-level zone emphasis  high gray-level zone emphasis  small distance low gray-level emphasis  small distance high gray-level emphasis  large distance low gray-level emphasis  large distance high gray-level emphasis  zone percentage  gray level variance  zone distance variance  zone distance entropy | gray-level non-uniformity  gray-level non-uniformity normalized  dependence count non-uniformity  dependence count non-uniformity normalized  low dependence emphasis  high dependence emphasis  low gray-level count emphasis  high gray-level count emphasis  low dependence low gray-level emphasis  low dependence high gray-level emphasis  high dependence low gray-level emphasis  high dependence high gray-level emphasis  gray level variance  dependence count variance  dependence count entropy  dependence count energy |

*Table 4: Feature types and names of extracted features in 2D- and 3D-radiomics.*

| **Dimension** | **Feature Aggregation Method** | **Abbreviation** | ***Example*** |
| --- | --- | --- | --- |
| *for GLCM and GLRLM:* | | | |
| 2D | no merging | (no prefix) |  |
|  | merging by direction | mbd | *(mbdGLCM_energy)* |
|  | merging by slice | mbs |  |
|  | full merging | mf |  |
| 3D | no merging | (no prefix) |  |
|  | full merging | M_ | *(M_GLCM_energy)* |
| *for GLSZM, GLDZM, NGTDM and NGLDM:* | | | |
| 2D | no merging | (no prefix) |  |
|  | merging | m | *(mGLSZM_energy)* |
| 3D | no merging | (no prefix) |  |

*Table 5: Naming of feature aggregation methods for texture features.*

| **3D** | **Prefix** | **2D** | **Prefix** |
| --- | --- | --- | --- |
| HHH | HHH_ | HH | HH_ |
| HHL | HHL_ |  |  |
| HLH | HLH_ | HL | HL_ |
| HLL | HLL_ |  |  |
| LHH | LHH_ | LH | LH_ |
| LHL | LHL_ |  |  |
| LLH | LLH_ | LL | LL_ |
| LLL | LLL_ |  |  |

*Table 6: Naming for wavelet transforms for features extracted in 2D and 3D.*

### Hyperparameters

In logistic regression, the hyperparameters used for tuning were:

- **penalty:** used to specify the norm used in the penalization (regularization),
- **C:** inverse of regularization strength,
- **l1_ratio:** elastic-net mixing parameter, which is used if penalty = ’elasticnet’,
- **class_weight:** weights associated with classes.

For extra trees, tuned hyperparameters were:

- **n_estimators:** number of trees in the forest,
- **criterion:** function to measure the quality of a split ("gini" for the Gini impurity and "entropy" for the information gain),
- **max_features:** maximum number of features considered for splitting a node,
- **min_samples_split:** minimum number of data points placed in a node before the node is split,
- **min_samples_leaf:** minimum number of data points required to be at a leaf node,
- **bootstrap:** method for sampling data points (with or without replacement),
- **class_weight:** weights associated with classes. [21]

|  | **Algorithm Hyperparameters Values** | | |
| --- | --- | --- | --- |
| **Feature selection** | Correlation  threshold | threshold | {’uniform’} |
|  |  | order | {’natural’, ’f-score’, ’h-score’} |
| **Feature selection**  **(based**  **on models)** | SFM-LR | penalty | {’l1’, ’l2’, ’elasticnet’} |
|  |  | C | {np.logspace(-4, 10, 1000, base=2)} |
|  |  | l1_ratio | {uniform()} |
|  |  | class_weight | {None, ’balanced’} |
|  | SFM-ET | n_estimators | {100, 200, 300, 500} |
|  |  | criterion | {’gini’, ’entropy’} |
|  |  | max_features | {np.arange(0.05, 1.01, 0.05)} |
|  |  | min_samples_split | {range(2,21)} |
|  |  | min_samples_leaf | {range(1,21)} |
|  |  | bootstrap | {True, False} |
|  |  | class_weight | {None, ’balanced’} |
| **Classification** | LR | penalty | {’l1’, ’l2’, ’elasticnet’} |
|  |  | C | {np.logspace(-5, 10, 1000, base=2)} |
|  |  | l1_ratio | {uniform()} |
|  |  | class_weight | {None, ’balanced’} |
|  | ET | n_estimators | {100, 200, 300, 500} |
|  |  | criterion | {’gini’, ’entropy’} |
|  |  | max_features | {np.arange(0.05, 1.01, 0.05)} |
|  |  | min_samples_split | {range(2,21)} |
|  |  | min_samples_leaf | {range(1,21)} |
|  |  | bootstrap | {True, False} |
|  |  | class_weight | {None, ’balanced’} |

*Table 7: Hyperparameters used for model building. For hyperparameters not mentioned here, scikit-learn’s default settings were used [27].*

### Models

Other ROC curves and PR curves for the best performing model per dimension and resolution.


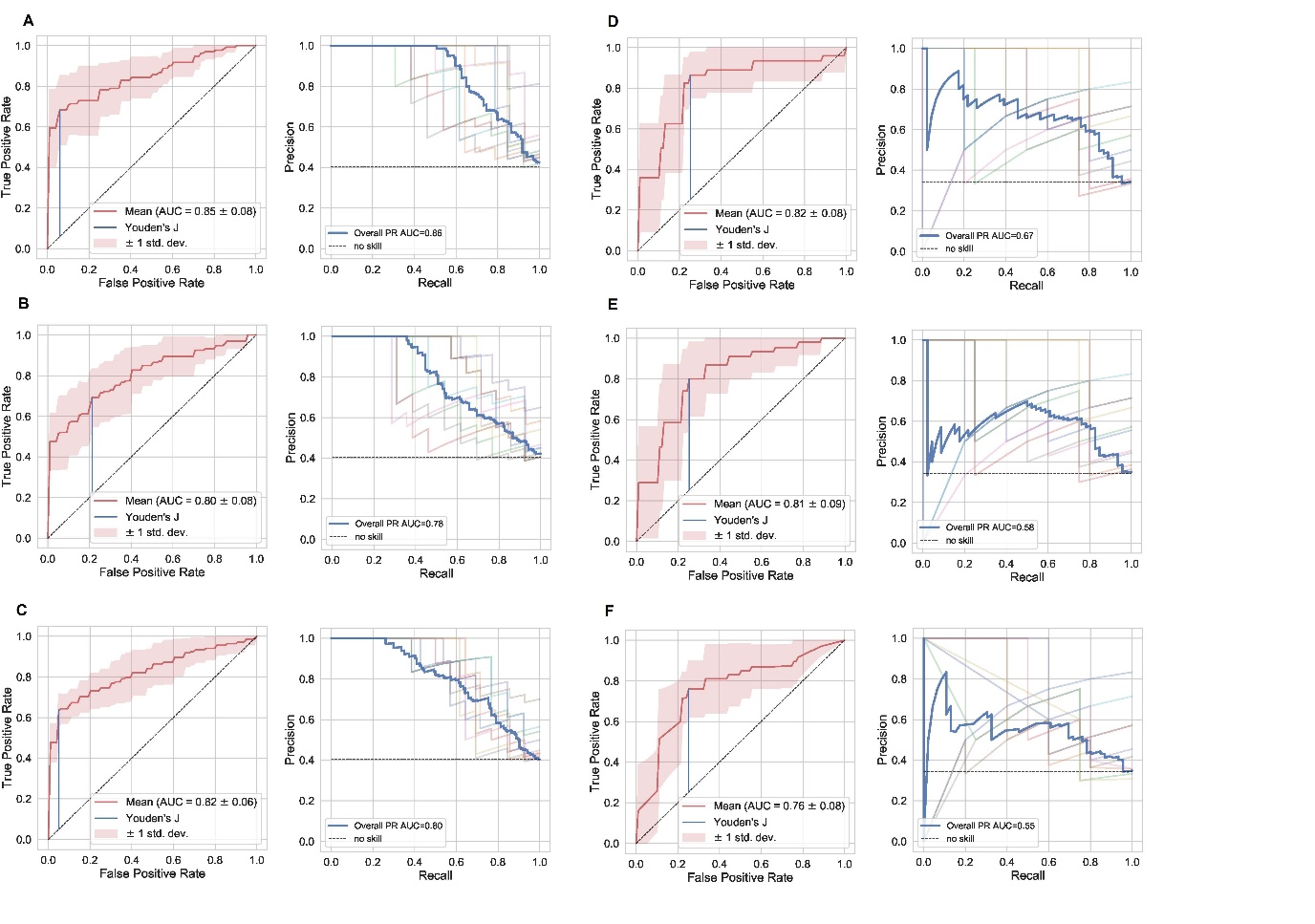
*Figure 6: ROC and PR curves of best performing models for diagnosis and staging. Diagnosis: A) 2D, 0.72 mm, B) 2D, 2 mm, C) 3D, 2 mm. Staging: D) 2D, 0.72 mm, E), 2D, 2 mm, F) 3D, 2 mm*

The partial dependence plots (PDP) of the best model for diagnosis are shown in Figure 7. Higher values in *GLCM-sumVariance* and *GLSZM-zsEntropy* features and lower values in the *LL-GLCM-clusterShade* feature indicated higher probability for classification as ILD.


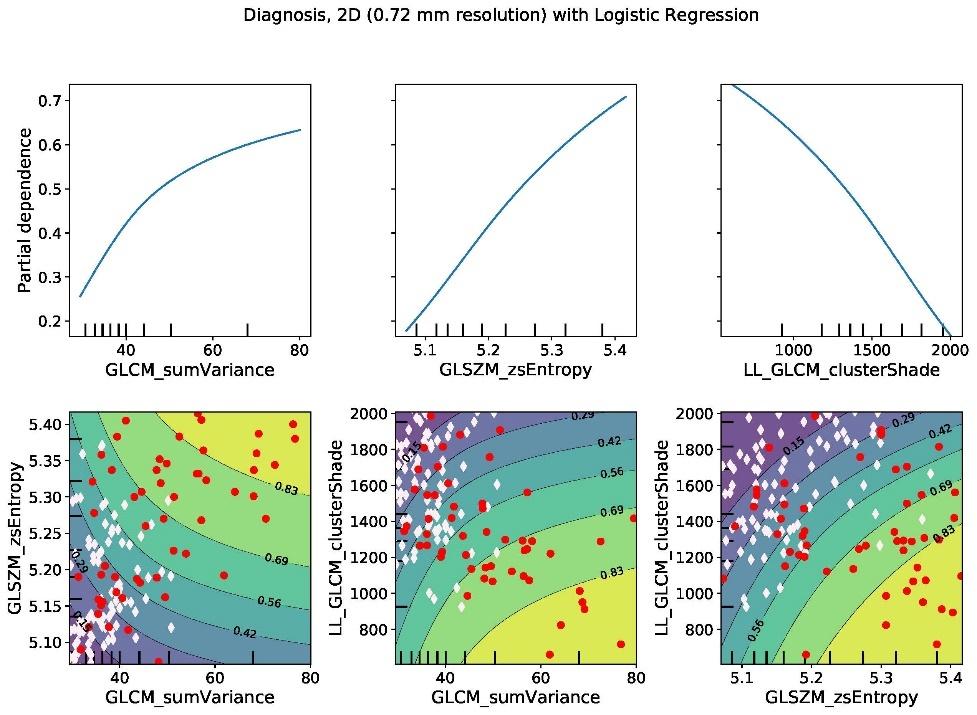


*Figure 7: Partial dependence plot for diagnosis in 2D (0.72 mm resolution) with logistic regression. White diamonds represent non-ILD patients. Red points stand for patients diseased with ILD.*

The partial dependence plots (PDP) of the best staging model are shown in Figure 8. The *skewness* and *kurtosis* features showed a negative correlation whereas the *standard deviation* feature (*hist_sd*) covered most range and correlated with extensive disease.


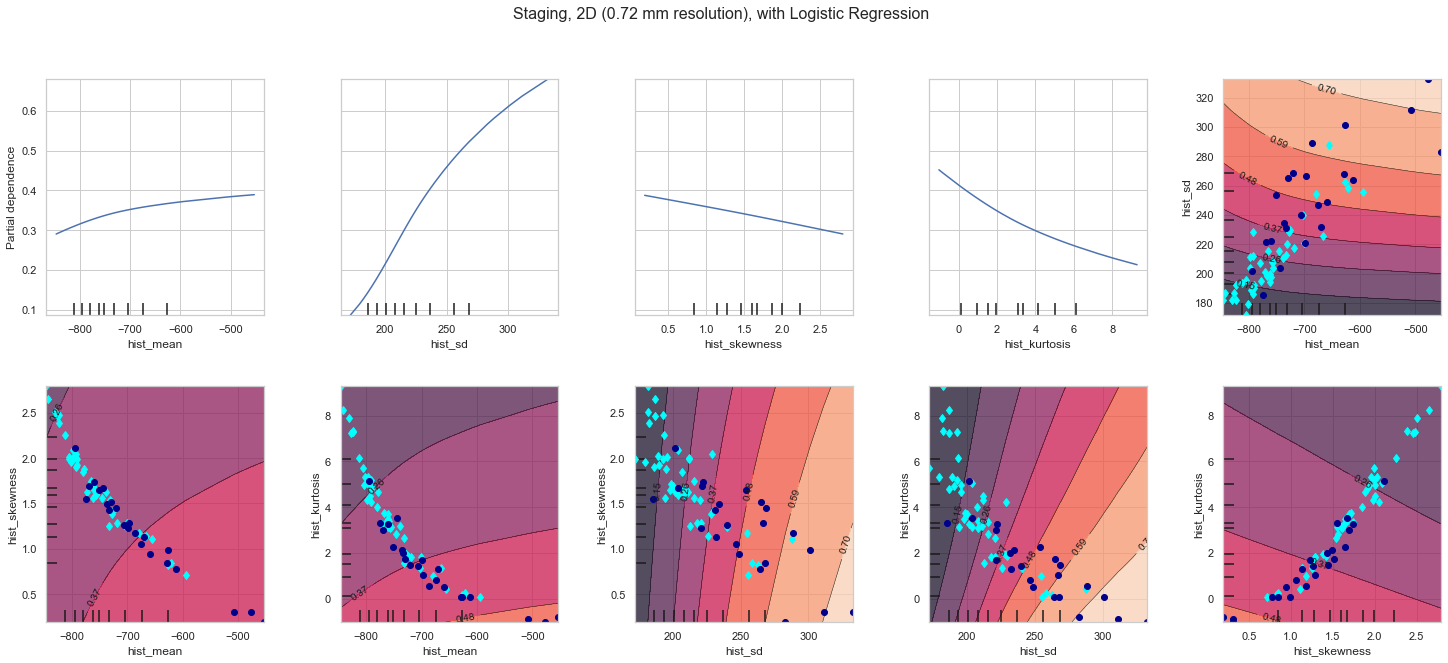


*Figure 8: Partial dependence plot for staging in 2D (0.72 mm), MSDSK-model. Cyan diamonds represent limited ILD patients. Blue points stand for extensive ILD patients.*
